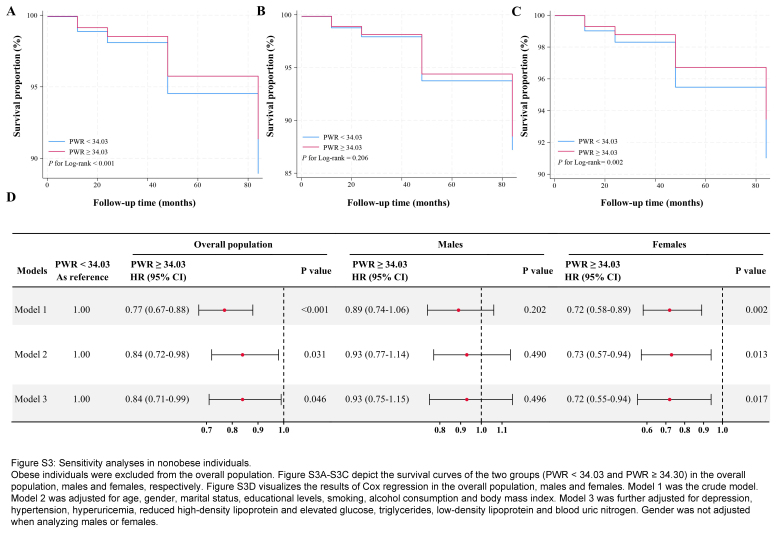# The independent predictive role of platelet to white blood cell ratio on all-cause mortality: a 7-year nationwide follow-up study in China

**DOI:** 10.1097/JS9.0000000000001688

**Published:** 2024-05-23

**Authors:** Xinyi Lyu, Yang Xiong, Lisha Jiang, Feng Qin

**Affiliations:** a Department of Urology, Institute of Urology, West China Hospital, Sichuan University; b Department of Urology and Andrology Laboratory, West China Hospital, Sichuan University; c Day Surgery Center, West China Hospital, Sichuan University, Sichuan Province, China

*Dear Editor,*

Emerging evidence suggests hematologic parameters like platelet to white blood cell ratio (PWR) serve as biomarkers for systemic inflammation, aiding disease diagnosis and prognosis evaluation^1,2^. A recent study published in the *International Journal of Surgery* constructed a risk prediction model for 6-month mortality after noncardiac surgery in elderly Chinese patients, incorporating factors like monocyte ratio and total blood cholesterol^3^. While some markers independently correlate with overall mortality, they lack cost-effectiveness and sample size validation^1^. Hence, identifying a novel, cost-effective marker is crucial for early disease intervention. PWR is computed from platelet and white blood cell counts^4^. However, its correlation with mortality in the general population remains unexplored. This study aims to investigate this relationship using data from the China Health and Retirement Longitudinal Study, involving 9359 participants. Survival status was assessed at baseline (2013) and follow-ups (2015, 2018), with a detailed inclusion process in Figure S1, Supplemental Digital Content 1, http://links.lww.com/JS9/C647. Participants were grouped based on the PWR median and baseline characteristics summarized (Table S1, Supplemental Digital Content 2, http://links.lww.com/JS9/C648). Multivariable Cox regression assessed the PWR-all-cause mortality relationship, adjusted for covariates, with subgroup, interaction, and sensitivity analyses conducted. For detailed statistical methods, refer to Supplementary files, Supplemental Digital Content 3, http://links.lww.com/JS9/C649.

The incidence rate of all-cause mortality was 10.38% for the PWR<34.03 group and 8.21% for the PWR ≥34.03 group (*P*<0.001, Table S1, Supplemental Digital Content 2). The survival curve indicated a superior survival probability for the group with PWR ≥34.03 compared to its counterpart with PWR<34.03 in the overall population (Fig. 1A, *P*<0.001). After stratified by gender, a higher survival probability was not found in males (Fig. 1B, *P*=0.137) but was identified in females (Fig. 1C, *P*<0.001). Further Cox regression models also supported the decreased risks of all-cause mortality for the PWR ≥34.03 group (Fig. 1D). In the overall population, the HRs were 0.75 [95% confidence interval (CI)=0.66−0.86, *P*<0.001], 0.84 (95% CI=0.72−0.97, *P*=0.021), and 0.85 (95% CI=0.73−0.99, *P*=0.038) in the crude, second, and full model, respectively. In males, these significant associations vanished (all *P*>0.05). Conversely, in females, associations strengthened, with HRs of 0.71 (95% CI=0.57−0.87, *P*=0.001), 0.73 (95% CI=0.58−0.92, *P*=0.008), and 0.73 (95% CI=0.56−0.94, *P*=0.015) in the crude, second, and full models, respectively. This evidence disclosed a significantly negative association between PWR and all-cause mortality in the overall population and females but not in males.

**Figure 1:**
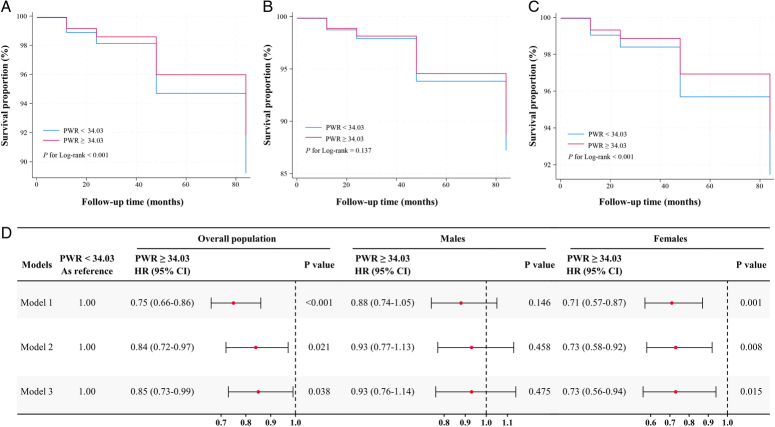
Association between PWR and all-cause mortality. (A–C) The survival curves of the two groups (PWR<34.03 and PWR ≥34.30) in the overall population, males and females, respectively. (D) Visualizes the results of Cox regression in the overall population, males and females. Model 1 was the crude model. Model 2 was adjusted for age, gender, marital status, educational levels, smoking, alcohol consumption, and BMI. Model 3 was further adjusted for depression, hypertension, hyperuricemia, reduced high-density lipoprotein and elevated glucose, triglycerides, low-density lipoprotein, and blood uric nitrogen. PWR, platelet to white blood cell ratio.

A series of subgroup analyses were performed (Figure S2, Supplemental Digital Content 4, http://links.lww.com/JS9/C650). Nearly all the HRs in the subgroups were <1, indicating a reduction in all-cause mortality risk for participants with PWR ≥34.03. In the studied subgroups, a notable reduction in all-cause mortality risk was observed in individuals aged 60 or above, females, individuals with a body mass index between 24.0 and 28.0 kg/m^2^, individuals with hypertension, elevated triglycerides, blood urea nitrogen ≤20 mg/dl, low-density lipoprotein ≤120 mg/dl, and absence of hyperuricemia (all *P*<0.05). The covariates exhibited no substantial influence on the correlation between PWR and overall mortality (all *P* for interaction>0.05).

Due to the adverse effects of obesity on health, obese individuals were excluded from the overall population (Figure S3A-S3C, Supplemental Digital Content 5, http://links.lww.com/JS9/C651). In both crude and full models (Figure S3D, Supplemental Digital Content 5, http://links.lww.com/JS9/C651), higher PWR was associated with higher survival probability in the overall population (*P*<0.001), and females (*P*=0.002) but not in males (*P*=0.202). Additionally, PWR was classified into quartiles and included in the regression model (Figure S4, Supplemental Digital Content 6, http://links.lww.com/JS9/C652). Higher PWR was linked to improved survival in the whole population, males, and females (all *P*<0.05, Figure S4A-S4C, Supplemental Digital Content 6, http://links.lww.com/JS9/C652). In the overall population (Figure S4D, Supplemental Digital Content 6, http://links.lww.com/JS9/C652), the Q4 group exhibited reduced all-cause mortality risk in both the crude and full models (all *P*<0.05). These associations were not replicated in males (*P*>0.05, Figure S4E, Supplemental Digital Content 6, http://links.lww.com/JS9/C652). The Q3 and Q4 groups had lower overall mortality risk than the Q1 group (Figure S4F, Supplemental Digital Content 6, http://links.lww.com/JS9/C652). The full models revealed HRs of 0.61 (95% CI=0.42−0.89, *P*<0.01) and 0.69 (95% CI=0.48−0.99, *P*<0.05) for the Q3 and Q4 groups, respectively.

Our study indicates that PWR is predictive of all-cause mortality. These findings suggest that PWR could potentially predict overall prognosis in the general population, aiding early identification and intervention. However, in the study by Wu *et al.*^3^, the hematologic risk indicator identified through their risk prediction model, mononuclear cell ratio, lacks validation from large epidemiological studies regarding its independent predictive role in mortality. Therefore, incorporating PWR as a candidate indicator in mortality prediction models may improve model optimization.

## Ethics approval

As all data is publicly accessible, institutional review board approval was not required.

## Consent

Written informed consent was obtained from the all the participants before attending this survey.

## Sources of funding

This work was supported by the Sichuan Science and Technology Program (No. 2022YFS0028), “1·3·5 Project for Disciplines of Excellence, West China Hospital, Sichuan University” (No. ZYGD23011). and the Frontiers Medical Center, Tianfu Jincheng Laboratory Foundation (Project No.TFJC2023010001).

## Author contribution

L.S.J., and F.Q.: conceptualization, data acquisition, and writing. X.Y.L.: statistical analysis and writing. Y.X.: review, editing, and supervision.

## Conflicts of interest disclosure

The authors declare no conflicts of interest.

## Research registration unique identifying number (UIN)

1. Name of the registry: not applicable.
2. Unique identifying number or registration ID: not applicable.
3. Hyperlink to your specific registration (must be publicly accessible and will be checked): not applicable.

## Guarantor

All authors.

## Data availability statement

Data are available from the corresponding author if the justification for the requirement is justified.

## Provenance and peer review

Not commissioned, externally peer-reviewed.

## Supplementary Material

**Figure:**
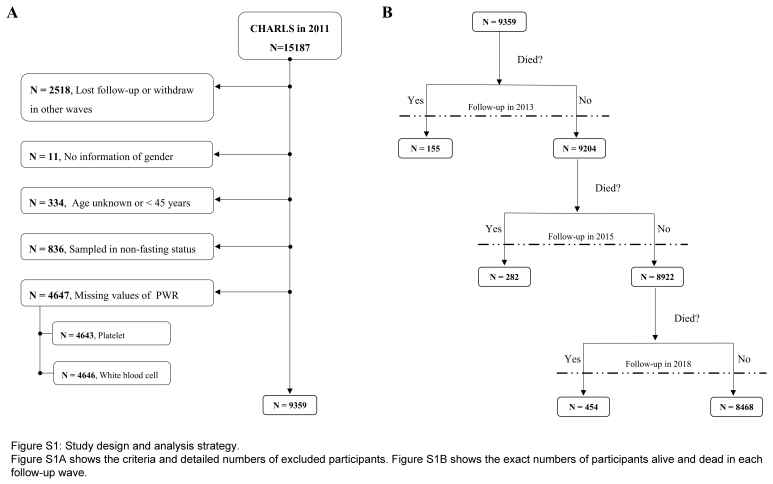


**Figure:**
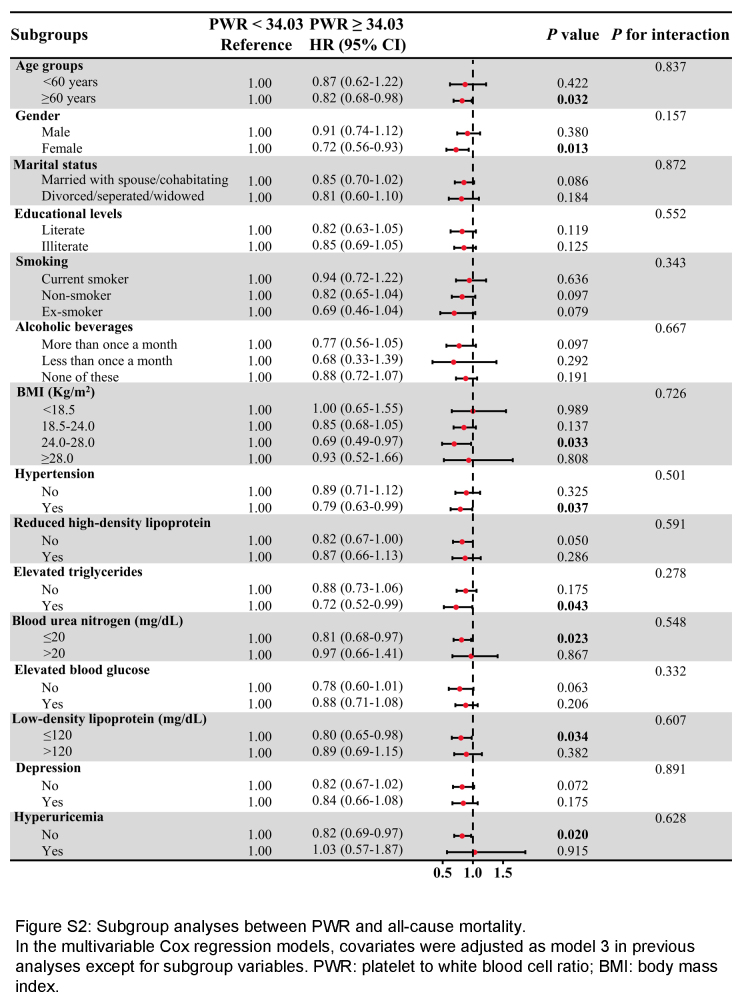


**Figure:**