# Supplementary material for: The independent predictive role of platelet to white blood cell ratio on all-cause mortality: a 7-year nationwide follow-up study in China
Source: Int J Surg. 2024 May 23;110(9):5923–5. doi: 10.1097/JS9.0000000000001688 (PMC11392087; doi:10.1097/JS9.0000000000001688)
Supplement: Supplementary file 1 [file js9-110-5923-s002.docx]

**Table S1:** **Characteristics of participants in the baseline survey.**

| **Characteristics** | **PWR < 34.03**  **N = 4681** | **PWR ≥ 34.03**  **N = 4678** | **Total**  **N = 9359** | ***P* value** |
| --- | --- | --- | --- | --- |
| Age (years) | 60.06 ± 9.61 | 58.94 ± 9.29 | 59.50 ± 9.47 | <0.001 |
| Gender |  |  |  |  |
| Male | 2485 (53.09%) | 1900 (40.62%) | 4385 (46.85%) | <0.001 |
| Female | 2196 (46.91%) | 2778 (59.38%) | 4974 (53.15%) |  |
| Marital status |  |  |  |  |
| Married with spouse/cohabitating | 3864 (82.55%) | 3924 (83.88%) | 7788 (83.21%) | 0.084 |
| Divorced/separated/widowed | 817 (17.45%) | 754 (16.12%) | 1571 (16.79%) |  |
| Educational levels |  |  |  |  |
| Literate | 2370 (50.63%) | 2477 (52.95%) | 4847 (51.79%) | 0.025 |
| Illiterate | 2311 (49.37%) | 2201 (47.05%) | 4512 (48.21%) |  |
| BMI (Kg/m^2^) |  |  |  |  |
| < 18.5 | 265 (6.65%) | 289 (7.18%) | 554 (6.92%) | 0.653 |
| 18.5-24.0 | 2099 (52.66%) | 2074 (51.54%) | 4173 (52.10%) |  |
| 24.0-28.0 | 1146 (28.75%) | 1184 (29.42%) | 2330 (29.09%) |  |
| ≥ 28.0 | 476 (11.94%) | 477 (11.85%) | 953 (11.90%) |  |
| Smoking |  |  |  |  |
| Current smoker | 1659 (35.52%) | 1244 (26.71%) | 2903 (31.12%) | <0.001 |
| Non-smoker | 2559 (54.78%) | 3041 (65.29%) | 5600 (60.03%) |  |
| Ex-smoker | 453 (9.70%) | 373 (8.01%) | 826 (8.85%) |  |
| Alcoholic Beverages |  |  |  |  |
| More than once a month | 1278 (27.37%) | 1094 (23.49%) | 2372 (25.43%) | <0.001 |
| Less than once a month | 392 (8.39%) | 336 (7.21%) | 728 (7.81%) |  |
| None of these | 3000 (64.24%) | 3227 (69.29%) | 6227 (66.76%) |  |
| Hypertension |  |  |  |  |
| No | 2428 (58.58%) | 2513 (60.06%) | 4941 (59.32%) | 0.168 |
| Yes | 1717 (41.42%) | 1671 (39.94%) | 3388 (40.68%) |  |
| Hyperuricemia |  |  |  |  |
| No | 4318 (93.22%) | 4405 (95.84%) | 8723 (94.53%) | <0.001 |
| Yes | 314 (6.78%) | 191 (4.16%) | 505 (5.47%) |  |
| Low-density lipoprotein (mg/dL) |  |  |  |  |
| ≤ 120 | 2685 (58.08%) | 2506 (54.66%) | 5191 (56.37%) | <0.001 |
| > 120 | 1938 (41.92%) | 2079 (45.34%) | 4017 (43.63%) |  |
| Reduced high-density lipoprotein |  |  |  |  |
| No | 2785 (60.06%) | 2628 (57.13%) | 5413 (58.60%) | 0.004 |
| Yes | 1852 (39.94%) | 1972 (42.87%) | 3824 (41.40%) |  |
| Elevated blood glucose |  |  |  |  |
| No | 1836 (39.68%) | 1962 (42.80%) | 3798 (41.23%) | 0.002 |
| Yes | 2791 (60.32%) | 2622 (57.20%) | 5413 (58.77%) |  |
| Elevated triglycerides |  |  |  |  |
| No | 3270 (70.58%) | 3278 (71.34%) | 6548 (70.96%) | 0.423 |
| Yes | 1363 (29.42%) | 1317 (28.66%) | 2680 (29.04%) |  |
| Blood urea nitrogen (mg/dL) |  |  |  |  |
| ≤ 20 | 3805 (82.20%) | 3987 (86.73%) | 7792 (84.46%) | <0.001 |
| > 20 | 824 (17.80%) | 610 (13.27%) | 1434 (15.54%) |  |
| Platelets (×10^9^/L) | 173.21 ± 56.57 | 250.90 ± 73.88 | 212.04 ± 76.41 | <0.001 |
| White blood cell (×10^9^/L) | 7.01 ± 2.54 | 5.50 ± 1.39 | 6.26 ± 2.18 | <0.001 |
| PWR ratio | 25.26 ± 6.12 | 46.66 ± 12.73 | 35.96 ± 14.63 | <0.001 |
| Death | 507 (10.38%) | 384 (8.21%) | 891 (9.52%) | <0.001 |

PWR: platelet to white blood cell ratio; BMI: body mass index.
