# Supplementary material for: The independent predictive role of platelet to white blood cell ratio on all-cause mortality: a 7-year nationwide follow-up study in China
Source: Int J Surg. 2024 May 23;110(9):5923–5. doi: 10.1097/JS9.0000000000001688 (PMC11392087; doi:10.1097/JS9.0000000000001688)
Supplement: Supplementary file 2 [file js9-110-5923-s003.docx]

**Materials and Methods**

***Study design***

A national longitudinal cohort from CHARLS was analyzed in this study. CHARLS is an ongoing survey aimed at the aging population (age ≥ 45 years) in China. Initiated in 2011, CHARLS sampled participants from 28 provinces covering 400 villages. The baseline participants underwent follow-ups every two or three years. To investigate the economic and health profiles of the aging population, the demographic characteristics, economic status, health status, medical history, physical examination and venous blood of the participants were collected. Detailed descriptions regarding the cohort profile, study design, sampling process and quality control of venous blood collection and determination can be found in previous studies^1,2^.

At baseline, 17,705 participants were sampled across China (**Figure S1A**). The participants were followed up in 2013, 2015 and 2018. The survival status was recorded. As shown in **Figure S1A**, participants who were lost to follow-up in the previous waves but were found to be dead in the next waves were excluded for the unclear time of death. Those participants cannot be included for further survival analysis. In addition, we also deleted participants lost to follow-up in 2018. In this step, 15,187 participants remained. As **Figure S1A** illustrates, participants with unclear gender, age, or aged < 45 years were further excluded. Given the impact of eating on blood biomarkers, participants with venous blood sampled in non-fasting status were excluded. In the final step, participants with missing values of PWR were excluded. Finally, the final analyses incorporated 9,359 participants. The number of deaths per follow-up is detailed in **Figure S1B**.

***Measurements of death and PWR***

The survival status was investigated in the baseline survey before the follow-up in 2013, 2015 and 2018. All deaths occurred in the follow-up waves and were confirmed by field investigation of interviewers. The researchers went to the residence of participants to have a face-to-face interview. If the participants died, the researcher collected related information from the household member who lived with the deceased. In CHARLS, there was no specific date of death. Thus, the time of death was recorded as the years of follow-up in this study.

PWR was calculated as the ratio of absolute platelet count to absolute white blood cell number^3^. Platelet and leukocyte quantifications were ascertained via automated complete blood count analysis. The analysis was conducted at local county health centers immediately after collection^2^.

***Measurements of hematological indicators***

To prevent mix-up of specimens, national identity cards and respondents’ photos were used to verify the identity of participants prior to the collection of venous blood. Fasting blood specimens were obtained by proficient medical staff from CHARLS or nurses from local medical institutions together according to a standard protocol^2^. To perform complete blood count analysis, 2 mL of whole blood was collected and then sent to a local county health center. The determination was made by automated analyzers. To determine other blood biomarkers, including lipids, glucose, uric acid and creatinine, 6 mL of whole blood was collected. The final determination was completed in the laboratory of Capital Medical University. The comprehensive methodology encompassing the collection of blood and determination of blood biomarkers can be referenced in a preceding publication^2^.

***Evaluation of covariates***

In this study, some covariates were evaluated and adjusted to assess the hazard ratio (HR) and 95% confidence interval (CI) between PWR and all-cause mortality. Demographic variables included age (years), gender, marital status (married/cohabitating or divorced/separated/widowed), and educational levels (literate and illiterate). Lifestyle variables included smoking (current, never and ex-smoker), alcohol intake (more than once a month, less than once a month and never) and body mass index (Kg/m^2^). Variables of disease status included depression (yes or no)^4^, hypertension (yes or no)^5^, hyperuricemia (yes or no)^6^, reduced HDL (yes or no)^7^, elevated triglycerides (yes or no)^7^, elevated blood glucose (yes or no)^7^, elevated LDL (yes or no)^8^ and elevated blood urea nitrogen (yes or no)^8^.

***Statistical analyses***

PWR was categorized based on the median, with categories being PWR < 34.03 and PWR ≥ 34.03. Descriptive statistics for continuous covariates were presented as mean ± standard error, and categorical variables were expressed as proportions. The relationship between PWR and all-cause mortality was evaluated using multivariable Cox regression. Three Cox regression models was established. No covariate was adjusted in model 1. In model 2, age, gender, educational levels, marital status, alcohol intake, smoking, and body mass index were included as covariates. Based on model 2, depression, hypertension, hyperuricemia, reduced HDL and elevated glucose, triglycerides, LDL and blood uric nitrogen were further adjusted in model 3. Subgroup and multiplicative interaction analyses were employed to scrutinize the interplay between PWR and covariates. To evaluate the differential survival probabilities, annual mortality data was converted to a monthly scale by a factor of 12. Subsequently, P values were derived using the log-rank test.

Two sensitivity analyses were conducted to verify the main findings. First, given the significant adverse effects of obesity on health, we excluded obese individuals (body mass index ≥ 28.0 Kg/m^2^) to verify the association in the non-obese participants. Second, we also stratified PWR according to quartiles (Q1, Q2, Q3 and Q4 groups) to verify the findings grouped according to the median.

Data analysis in this study was conducted using Stata 16.0 (Stata Corporation, TX, USA), with a two-sided *P* value of less than 0.05 denoting statistical significance.

**References**

1. Zhao Y, Hu Y, Smith JP, Strauss J, Yang G. Cohort profile: the China Health and Retirement Longitudinal Study (CHARLS). *Int J Epidemiol* 2014;43:61–8.

2. Chen X, Crimmins E, Hu PP, Kim JK, Meng Q, Strauss J, Wang Y, Zeng J, Zhang Y, Zhao Y. Venous Blood-Based Biomarkers in the China Health and Retirement Longitudinal Study: Rationale, Design, and Results From the 2015 Wave. *Am J Epidemiol* 2019;188:1871–7.

3. Zhao S, Pan H, Guo Q, Xie W, Wang J. Platelet to white blood cell ratio was an independent prognostic predictor in acute myeloid leukemia. *Hematology* 2022;27:426–30.

4. Xiong Y, Zhang Y-C, Jin T, Qin F, Yuan J-H. Depressive males have higher odds of lower urinary tract symptoms suggestive of benign prostatic hyperplasia: a retrospective cohort study based on propensity score matching. *Asian J Androl* 2021;23:633–9.

5. Poulter NR, Prabhakaran D, Caulfield M. Hypertension. *Lancet* 2015;386:801–12.

6. Piao W, Zhao L, Yang Y, Fang H, Ju L, Cai S, Yu D. The Prevalence of Hyperuricemia and Its Correlates among Adults in China: Results from CNHS 2015-2017. *Nutrients* 2022;14:4095.

7. Shi Z, Tuomilehto J, Kronfeld-Schor N, Alberti GK, Stern N, El-Osta A, Bilu C, Einat H, Zimmet P. The circadian syndrome predicts cardiovascular disease better than metabolic syndrome in Chinese adults. *J Intern Med* 2021;289:851–60.

8. Xiong Y, Zhang Y, Zhang F, Wu C, Qin F, Yuan J. Prevalence and associated factors of metabolic syndrome in Chinese middle-aged and elderly population: a national cross-sectional study. *Aging Male* 2021;24:148–59.
